# 2,2′-[(4-Eth­oxy­phen­yl)methyl­ene]bis­(3-hy­droxy-5,5-dimethyl­cyclo­hex-2-en-1-one)

**DOI:** 10.1107/S1600536812033934

**Published:** 2012-08-04

**Authors:** N. Sureshbabu, V. Sughanya

**Affiliations:** a Department of Chemistry, Annamalai University, Annamalai Nagar 608 002, Tamil Nadu, India

## Abstract

In the title compound, C_25_H_32_O_5_, the two cyclo­hexenone rings have envelope conformations with the C atom bearing two methyl groups as the flap atom in each ring. Relatively strong intra­molecular O—H⋯O hydrogen bonds are observed.

## Related literature

For the synthesis of bis­dimedones, see: Vang & Stankevich (1960 ▶); Hilderbrand & Weissleder (2007 ▶). For their pharmaceutical properties, see: Lambert *et al.* (1997 ▶); Poupelin *et al.* (1978 ▶); Hideo (1981 ▶); Selvanayagam *et al.* (1996 ▶); Jonathan *et al.* (1988 ▶). For the crystal structures of related xanthene derivatives, see: Odabaşoğlu *et al.* (2008 ▶); Mehdi *et al.* (2011 ▶); Ravikumar *et al.* (2012 ▶). For the assignment of ring conformations, see: Cremer & Pople (1975 ▶).
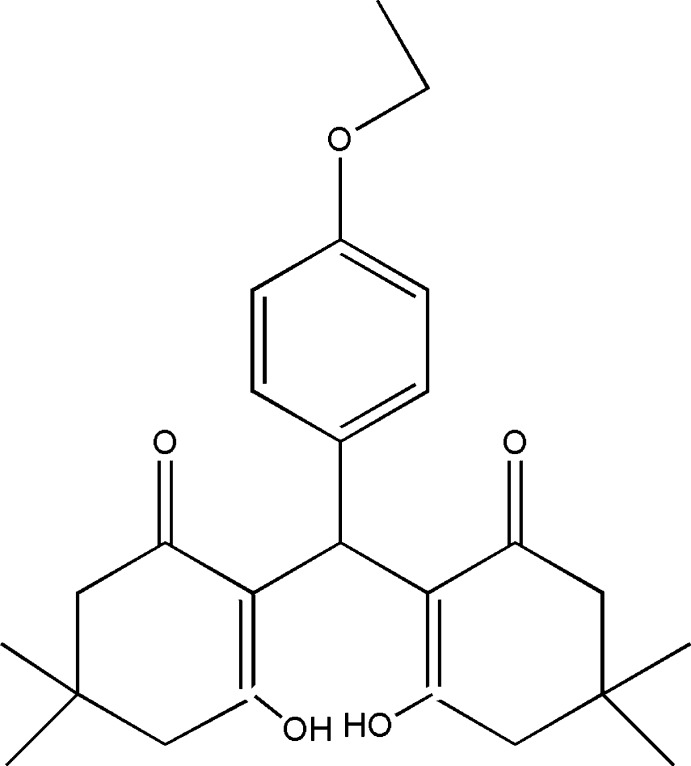


## Experimental

### 

#### Crystal data

C_25_H_32_O_5_
*M* *_r_* = 412.51
Monoclinic, 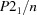

*a* = 9.774 (5) Å
*b* = 10.698 (5) Å
*c* = 21.578 (5) Å
β = 93.735 (5)°
*V* = 2251.5 (16) Å^3^
*Z* = 4
Mo *K*α radiation
μ = 0.08 mm^−1^
*T* = 293 K
0.30 × 0.20 × 0.20 mm

#### Data collection

Bruker Kappa APEXII CCD diffractometer
Absorption correction: multi-scan (*SADABS*; Bruker, 2004) ▶ *T*_min_ = 0.961, *T*_max_ = 0.989
17055 measured reflections
3962 independent reflections
3065 reflections with *I* > 2σ(*I*)
*R*_int_ = 0.028

#### Refinement

*R*[*F* ^2^ > 2σ(*F* ^2^)] = 0.042
*wR*(*F* ^2^) = 0.122
*S* = 1.03
3962 reflections
272 parameters
H-atom parameters constrained
Δρ_max_ = 0.24 e Å^−3^
Δρ_min_ = −0.23 e Å^−3^

### 

Data collection: *APEX2* (Bruker, 2004 ▶); cell refinement: *APEX2* and *SAINT* (Bruker, 2004 ▶); data reduction: *SAINT* and *XPREP* (Bruker, 2004 ▶); program(s) used to solve structure: *SIR92* (Altomare *et al.*, 1993 ▶); program(s) used to refine structure: *SHELXL97* (Sheldrick, 2008 ▶); molecular graphics: *ORTEP-3* (Farrugia, 1997 ▶) and *Mercury* (Macrae *et al.*, 2008 ▶); software used to prepare material for publication: *SHELXL97*.

## Supplementary Material

Crystal structure: contains datablock(s) global, I. DOI: 10.1107/S1600536812033934/wn2485sup1.cif

Structure factors: contains datablock(s) I. DOI: 10.1107/S1600536812033934/wn2485Isup2.hkl

Additional supplementary materials:  crystallographic information; 3D view; checkCIF report

## Figures and Tables

**Table 1:** Hydrogen-bond geometry (Å, °)

*D*—H⋯*A*	*D*—H	H⋯*A*	*D*⋯*A*	*D*—H⋯*A*
O3—H3*A*⋯O5	0.82	1.83	2.631 (2)	164
O4—H4*A*⋯O2	0.82	1.78	2.5864 (19)	167

**Fig. 1.**
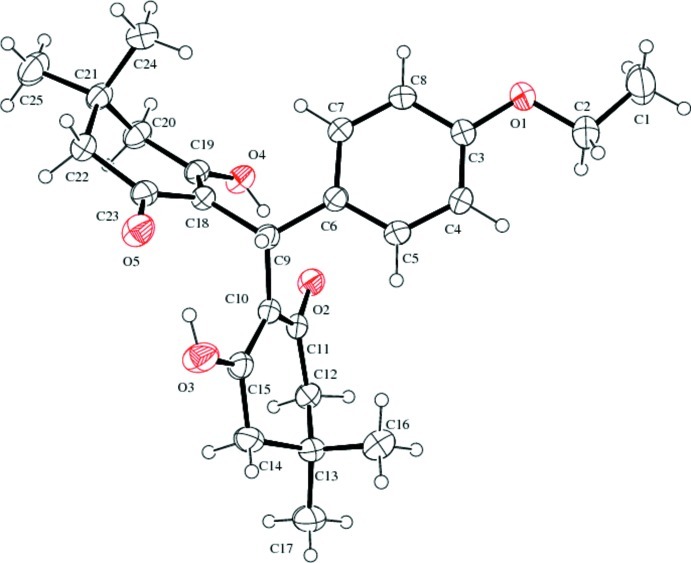
A view of the structure of the title compound. Displacement ellipsoids are drawn at the 50% probability level. Hydrogen atoms are shown as spheres of arbitrary radius.

**Fig. 2.**
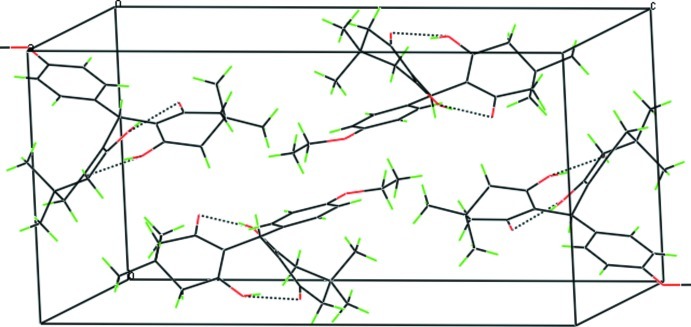
A view of the packing in the crystal structure, showing intramolecular O—H···O hydrogen bonds as dotted lines.

## supplementary crystallographic information

### Comment

Several methods have been reported in the literature (Vang & Stankevich, 1960; Hilderbrand & Weissleder, 2007) for the synthesis of the title compound. Xanthene derivatives possess biological properties such as antibacterial, antiviral and anti-inflammatory activities (Jonathan *et al.*, 1988) and are therefore used in medicine.

In the title compound, the cyclohexenone rings C10–C15 and C18–C23 both adopt envelope conformations, with flap atoms C13 and C21, respectively.The dihedral angle between the two cyclohexenone planes Q(C10/C11/C12/C14/C15) and R(C18/C19/C20/C22/C23) is 58.42 (3)°. The dihedral angle between the benzene ring P(C3–C8) and cyclohexenone planes Q and R are 66.37 (3)° and 61.41 (4)°, respectively.. The hydroxy and carbonyl oxygen atoms face each other and are oriented to allow for the formation of two intramolecular O—H···O hydrogen bonds (Table 1, Fig. 2), typical for xanthene derivatives.

### Experimental

The title compound was prepared in a single stage. A mixture of 4-Ethoxybenzaldehyde (1.2g, 8 mmol), 5,5-dimethylcyclohexane-1,3-dione (2.24g, 16 mmol) and 20ml of ethanol was heated to 70 °C for about 10 minutes. The reaction mixture was allowed to cool to room temperature and the resulting title compound, 2,2'-((4-ethoxyphenyl)methylene) bis(3-hydroxy-5,5-dimethylcyclohex-2-enone) was filtered and dried (M.p. 411 K; Yield 78% ).

### Refinement

All hydrogen atoms were identified from difference electron density peaks and subsequently treated as riding atoms with d(Csp^2^—H) = 0.93 Å, d(Cmethyl—H) = 0.96 Å, d(Cmethylene—H) = 0.97 Å, d(Cmethine—H) = 0.98 Å; d(O—H) = 0.82 Å; U_iso_(H) = xU_eq_(C,O), where x = 1.5 for methyl H and 1.2 for all other H atoms.

### Crystal data

**Table:**

C_25_H_32_O_5_	*F*(000) = 888
*M**_r_* = 412.51	*D*_x_ = 1.217 Mg m^−^^3^
Monoclinic, *P*2_1_/*n*	Mo *K*α radiation, λ = 0.71073 Å
*a* = 9.774 (5) Å	Cell parameters from 6149 reflections
*b* = 10.698 (5) Å	θ = 2.1–27.1°
*c* = 21.578 (5) Å	µ = 0.08 mm^−^^1^
β = 93.735 (5)°	*T* = 293 K
*V* = 2251.5 (16) Å^3^	Block, colourless
*Z* = 4	0.30 × 0.20 × 0.20 mm

### Data collection

**Table:**

Bruker Kappa APEXII CCD diffractometer	3962 independent reflections
Radiation source: fine-focus sealed tube	3065 reflections with *I* > 2σ(*I*)
Graphite monochromator	*R*_int_ = 0.028
ω and φ scan	θ_max_ = 25.0°, θ_min_ = 2.1°
Absorption correction: multi-scan (*SADABS*; Bruker, 2005)	*h* = −11→7
*T*_min_ = 0.961, *T*_max_ = 0.989	*k* = −12→12
17055 measured reflections	*l* = −24→25

### Refinement

**Table:**

Refinement on *F*^2^	Secondary atom site location: difference Fourier map
Least-squares matrix: full	Hydrogen site location: inferred from neighbouring sites
*R*[*F*^2^ > 2σ(*F*^2^)] = 0.042	H-atom parameters constrained
*wR*(*F*^2^) = 0.122	*w* = 1/[σ^2^(*F*_o_^2^) + (0.0573*P*)^2^ + 0.7618*P*] where *P* = (*F*_o_^2^ + 2*F*_c_^2^)/3
*S* = 1.03	(Δ/σ)_max_ < 0.001
3962 reflections	Δρ_max_ = 0.24 e Å^−^^3^
272 parameters	Δρ_min_ = −0.23 e Å^−^^3^
0 restraints	Extinction correction: *SHELXL97* (Sheldrick, 2008), Fc^*^=kFc[1+0.001xFc^2^λ^3^/sin(2θ)]^-1/4^
Primary atom site location: structure-invariant direct methods	Extinction coefficient: 0.0143 (14)

### Special details

**Table:**

Geometry. All e.s.d.'s (except the e.s.d. in the dihedral angle between two l.s. planes) are estimated using the full covariance matrix. The cell e.s.d.'s are taken into account individually in the estimation of e.s.d.'s in distances, angles and torsion angles; correlations between e.s.d.'s in cell parameters are only used when they are defined by crystal symmetry. An approximate (isotropic) treatment of cell e.s.d.'s is used for estimating e.s.d.'s involving l.s. planes.
Refinement. Refinement of *F*^2^ against ALL reflections. The weighted *R*-factor *wR* and goodness of fit *S* are based on *F*^2^, conventional *R*-factors *R* are based on *F*, with *F* set to zero for negative *F*^2^. The threshold expression of *F*^2^ > σ(*F*^2^) is used only for calculating *R*-factors(gt) *etc*. and is not relevant to the choice of reflections for refinement. *R*-factors based on *F*^2^ are statistically about twice as large as those based on *F*, and *R*- factors based on ALL data will be even larger.

### Fractional atomic coordinates and isotropic or equivalent isotropic displacement parameters (Å^2^)

**Table:**

	*x*	*y*	*z*	*U*_iso_*/*U*_eq_	
C1	0.1136 (3)	0.5154 (3)	0.33826 (11)	0.0776 (7)	
H1A	0.1550	0.5226	0.2993	0.116*	
H1B	0.0836	0.5964	0.3510	0.116*	
H1C	0.0363	0.4600	0.3337	0.116*	
C2	0.2151 (2)	0.4651 (3)	0.38572 (10)	0.0707 (7)	
H2A	0.2457	0.3831	0.3732	0.085*	
H2B	0.2943	0.5198	0.3900	0.085*	
C3	0.22806 (19)	0.40529 (19)	0.49280 (8)	0.0495 (5)	
C4	0.3642 (2)	0.3757 (2)	0.49343 (9)	0.0567 (6)	
H4	0.4129	0.3911	0.4586	0.068*	
C5	0.42951 (19)	0.32300 (19)	0.54571 (9)	0.0518 (5)	
H5	0.5221	0.3032	0.5452	0.062*	
C6	0.36275 (17)	0.29861 (16)	0.59866 (7)	0.0368 (4)	
C7	0.22621 (18)	0.33226 (18)	0.59738 (8)	0.0474 (5)	
H7	0.1781	0.3191	0.6326	0.057*	
C8	0.15904 (19)	0.3846 (2)	0.54565 (9)	0.0547 (5)	
H8	0.0669	0.4061	0.5463	0.066*	
C9	0.44542 (16)	0.24953 (15)	0.65658 (7)	0.0350 (4)	
H9	0.4980	0.3224	0.6720	0.042*	
C10	0.55496 (16)	0.15470 (16)	0.64172 (7)	0.0359 (4)	
C11	0.52716 (17)	0.05495 (16)	0.60035 (7)	0.0373 (4)	
C12	0.63981 (18)	−0.02547 (18)	0.57850 (8)	0.0474 (5)	
H12A	0.6192	−0.0451	0.5350	0.057*	
H12B	0.6408	−0.1035	0.6014	0.057*	
C13	0.78274 (18)	0.0323 (2)	0.58573 (8)	0.0497 (5)	
C14	0.80122 (19)	0.0814 (2)	0.65212 (9)	0.0564 (5)	
H14A	0.8101	0.0108	0.6803	0.068*	
H14B	0.8858	0.1288	0.6566	0.068*	
C15	0.68583 (18)	0.16245 (18)	0.67054 (8)	0.0433 (4)	
C16	0.7976 (2)	0.1388 (2)	0.53921 (10)	0.0651 (6)	
H16A	0.8883	0.1733	0.5446	0.098*	
H16B	0.7314	0.2027	0.5462	0.098*	
H16C	0.7825	0.1071	0.4977	0.098*	
C17	0.8898 (2)	−0.0678 (2)	0.57416 (11)	0.0714 (7)	
H17A	0.8820	−0.1349	0.6033	0.107*	
H17B	0.9798	−0.0319	0.5793	0.107*	
H17C	0.8746	−0.0994	0.5326	0.107*	
C18	0.36132 (16)	0.21294 (15)	0.71075 (7)	0.0341 (4)	
C19	0.25324 (17)	0.12859 (16)	0.70621 (8)	0.0373 (4)	
C20	0.1721 (2)	0.09839 (17)	0.76079 (9)	0.0472 (5)	
H20A	0.2115	0.0251	0.7815	0.057*	
H20B	0.0790	0.0777	0.7459	0.057*	
C21	0.16771 (18)	0.20449 (17)	0.80774 (8)	0.0434 (4)	
C22	0.31497 (19)	0.24452 (19)	0.82458 (8)	0.0457 (5)	
H22A	0.3135	0.3233	0.8468	0.055*	
H22B	0.3576	0.1829	0.8526	0.055*	
C23	0.40218 (18)	0.25987 (16)	0.77023 (8)	0.0394 (4)	
C24	0.08261 (19)	0.31254 (19)	0.78021 (9)	0.0516 (5)	
H24A	0.0811	0.3791	0.8100	0.077*	
H24B	−0.0093	0.2845	0.7698	0.077*	
H24C	0.1224	0.3422	0.7434	0.077*	
C25	0.1019 (2)	0.1593 (2)	0.86609 (10)	0.0667 (6)	
H25A	0.1000	0.2266	0.8954	0.100*	
H25B	0.1543	0.0912	0.8842	0.100*	
H25C	0.0099	0.1318	0.8552	0.100*	
O1	0.15317 (15)	0.45642 (16)	0.44304 (6)	0.0703 (5)	
O2	0.40539 (12)	0.02575 (12)	0.58061 (5)	0.0467 (3)	
O3	0.71819 (13)	0.23817 (14)	0.71624 (6)	0.0597 (4)	
H3A	0.6481	0.2663	0.7299	0.072*	
O4	0.21555 (12)	0.06855 (12)	0.65595 (6)	0.0477 (3)	
H4A	0.2793	0.0663	0.6331	0.057*	
O5	0.51622 (13)	0.31629 (13)	0.78119 (6)	0.0527 (4)	

### Atomic displacement parameters (Å^2^)

**Table:**

	*U*^11^	*U*^22^	*U*^33^	*U*^12^	*U*^13^	*U*^23^
C1	0.0780 (16)	0.0951 (18)	0.0589 (14)	−0.0061 (14)	−0.0029 (12)	0.0242 (13)
C2	0.0646 (14)	0.0954 (18)	0.0529 (13)	0.0138 (12)	0.0107 (11)	0.0272 (13)
C3	0.0478 (11)	0.0603 (12)	0.0411 (10)	0.0145 (9)	0.0076 (8)	0.0138 (9)
C4	0.0508 (12)	0.0773 (14)	0.0440 (11)	0.0176 (10)	0.0187 (9)	0.0229 (10)
C5	0.0379 (10)	0.0693 (13)	0.0497 (11)	0.0141 (9)	0.0138 (8)	0.0178 (10)
C6	0.0370 (9)	0.0376 (9)	0.0365 (9)	0.0018 (7)	0.0066 (7)	0.0024 (7)
C7	0.0406 (10)	0.0626 (12)	0.0405 (10)	0.0109 (9)	0.0132 (8)	0.0102 (9)
C8	0.0390 (10)	0.0751 (14)	0.0510 (11)	0.0181 (9)	0.0117 (8)	0.0158 (10)
C9	0.0330 (8)	0.0373 (9)	0.0349 (9)	−0.0032 (7)	0.0035 (7)	−0.0011 (7)
C10	0.0345 (9)	0.0440 (10)	0.0294 (8)	0.0006 (7)	0.0034 (7)	0.0028 (7)
C11	0.0385 (9)	0.0458 (10)	0.0278 (8)	0.0013 (7)	0.0036 (7)	0.0048 (7)
C12	0.0498 (11)	0.0536 (11)	0.0393 (10)	0.0099 (9)	0.0070 (8)	−0.0028 (9)
C13	0.0400 (10)	0.0690 (13)	0.0404 (10)	0.0151 (9)	0.0056 (8)	0.0012 (9)
C14	0.0379 (10)	0.0842 (15)	0.0464 (11)	0.0132 (10)	−0.0034 (8)	−0.0067 (11)
C15	0.0369 (9)	0.0597 (11)	0.0334 (9)	−0.0015 (8)	0.0019 (7)	−0.0024 (9)
C16	0.0528 (13)	0.0821 (16)	0.0623 (13)	0.0027 (11)	0.0171 (10)	0.0066 (12)
C17	0.0564 (13)	0.0975 (18)	0.0607 (13)	0.0304 (12)	0.0077 (10)	−0.0055 (13)
C18	0.0346 (9)	0.0354 (9)	0.0325 (9)	0.0014 (7)	0.0043 (7)	−0.0005 (7)
C19	0.0379 (9)	0.0359 (9)	0.0384 (9)	0.0019 (7)	0.0056 (7)	−0.0020 (8)
C20	0.0492 (11)	0.0432 (10)	0.0509 (11)	−0.0054 (8)	0.0168 (8)	0.0013 (9)
C21	0.0460 (10)	0.0459 (10)	0.0397 (10)	0.0004 (8)	0.0142 (8)	0.0002 (8)
C22	0.0494 (11)	0.0536 (11)	0.0347 (10)	0.0085 (9)	0.0067 (8)	−0.0011 (8)
C23	0.0386 (9)	0.0394 (9)	0.0405 (10)	0.0030 (8)	0.0048 (7)	−0.0019 (8)
C24	0.0437 (11)	0.0551 (12)	0.0569 (12)	0.0061 (9)	0.0096 (9)	−0.0031 (10)
C25	0.0746 (15)	0.0732 (15)	0.0559 (13)	−0.0010 (12)	0.0305 (11)	0.0050 (11)
O1	0.0564 (9)	0.1055 (13)	0.0500 (8)	0.0307 (8)	0.0115 (7)	0.0312 (8)
O2	0.0411 (7)	0.0590 (8)	0.0398 (7)	−0.0045 (6)	0.0008 (5)	−0.0075 (6)
O3	0.0373 (7)	0.0860 (10)	0.0556 (8)	−0.0051 (7)	0.0016 (6)	−0.0234 (8)
O4	0.0441 (7)	0.0527 (8)	0.0472 (7)	−0.0104 (6)	0.0099 (6)	−0.0117 (6)
O5	0.0459 (8)	0.0660 (9)	0.0460 (7)	−0.0096 (6)	0.0029 (6)	−0.0164 (7)

### Geometric parameters (Å, º)

**Table:**

C1—C2	1.481 (3)	C14—C15	1.497 (3)
C1—H1A	0.9600	C14—H14A	0.9700
C1—H1B	0.9600	C14—H14B	0.9700
C1—H1C	0.9600	C15—O3	1.299 (2)
C2—O1	1.415 (2)	C16—H16A	0.9600
C2—H2A	0.9700	C16—H16B	0.9600
C2—H2B	0.9700	C16—H16C	0.9600
C3—C4	1.367 (3)	C17—H17A	0.9600
C3—O1	1.373 (2)	C17—H17B	0.9600
C3—C8	1.380 (3)	C17—H17C	0.9600
C4—C5	1.380 (3)	C18—C19	1.388 (2)
C4—H4	0.9300	C18—C23	1.411 (2)
C5—C6	1.377 (2)	C19—O4	1.293 (2)
C5—H5	0.9300	C19—C20	1.497 (2)
C6—C7	1.381 (2)	C20—C21	1.524 (3)
C6—C9	1.536 (2)	C20—H20A	0.9700
C7—C8	1.377 (3)	C20—H20B	0.9700
C7—H7	0.9300	C21—C24	1.522 (3)
C8—H8	0.9300	C21—C22	1.523 (3)
C9—C18	1.523 (2)	C21—C25	1.529 (3)
C9—C10	1.524 (2)	C22—C23	1.503 (2)
C9—H9	0.9800	C22—H22A	0.9700
C10—C15	1.388 (2)	C22—H22B	0.9700
C10—C11	1.406 (2)	C23—O5	1.276 (2)
C11—O2	1.276 (2)	C24—H24A	0.9600
C11—C12	1.497 (2)	C24—H24B	0.9600
C12—C13	1.526 (3)	C24—H24C	0.9600
C12—H12A	0.9700	C25—H25A	0.9600
C12—H12B	0.9700	C25—H25B	0.9600
C13—C14	1.525 (3)	C25—H25C	0.9600
C13—C17	1.529 (3)	O3—H3A	0.8200
C13—C16	1.531 (3)	O4—H4A	0.8200
			
C2—C1—H1A	109.5	H14A—C14—H14B	107.7
C2—C1—H1B	109.5	O3—C15—C10	123.30 (17)
H1A—C1—H1B	109.5	O3—C15—C14	114.36 (15)
C2—C1—H1C	109.5	C10—C15—C14	122.33 (16)
H1A—C1—H1C	109.5	C13—C16—H16A	109.5
H1B—C1—H1C	109.5	C13—C16—H16B	109.5
O1—C2—C1	108.82 (19)	H16A—C16—H16B	109.5
O1—C2—H2A	109.9	C13—C16—H16C	109.5
C1—C2—H2A	109.9	H16A—C16—H16C	109.5
O1—C2—H2B	109.9	H16B—C16—H16C	109.5
C1—C2—H2B	109.9	C13—C17—H17A	109.5
H2A—C2—H2B	108.3	C13—C17—H17B	109.5
C4—C3—O1	124.68 (16)	H17A—C17—H17B	109.5
C4—C3—C8	118.85 (17)	C13—C17—H17C	109.5
O1—C3—C8	116.46 (16)	H17A—C17—H17C	109.5
C3—C4—C5	120.04 (17)	H17B—C17—H17C	109.5
C3—C4—H4	120.0	C19—C18—C23	117.68 (15)
C5—C4—H4	120.0	C19—C18—C9	124.00 (15)
C6—C5—C4	122.47 (17)	C23—C18—C9	118.13 (14)
C6—C5—H5	118.8	O4—C19—C18	123.79 (15)
C4—C5—H5	118.8	O4—C19—C20	114.57 (15)
C5—C6—C7	116.33 (16)	C18—C19—C20	121.63 (15)
C5—C6—C9	119.16 (15)	C19—C20—C21	113.67 (15)
C7—C6—C9	124.19 (15)	C19—C20—H20A	108.8
C8—C7—C6	122.12 (16)	C21—C20—H20A	108.8
C8—C7—H7	118.9	C19—C20—H20B	108.8
C6—C7—H7	118.9	C21—C20—H20B	108.8
C7—C8—C3	120.15 (17)	H20A—C20—H20B	107.7
C7—C8—H8	119.9	C24—C21—C22	111.33 (16)
C3—C8—H8	119.9	C24—C21—C20	110.15 (16)
C18—C9—C10	114.33 (14)	C22—C21—C20	107.52 (15)
C18—C9—C6	115.51 (13)	C24—C21—C25	108.22 (16)
C10—C9—C6	113.32 (13)	C22—C21—C25	109.52 (16)
C18—C9—H9	103.9	C20—C21—C25	110.09 (16)
C10—C9—H9	103.9	C23—C22—C21	114.81 (14)
C6—C9—H9	103.9	C23—C22—H22A	108.6
C15—C10—C11	117.65 (16)	C21—C22—H22A	108.6
C15—C10—C9	120.37 (15)	C23—C22—H22B	108.6
C11—C10—C9	121.95 (14)	C21—C22—H22B	108.6
O2—C11—C10	122.34 (15)	H22A—C22—H22B	107.5
O2—C11—C12	116.30 (16)	O5—C23—C18	121.93 (16)
C10—C11—C12	121.34 (15)	O5—C23—C22	116.10 (15)
C11—C12—C13	114.95 (16)	C18—C23—C22	121.96 (16)
C11—C12—H12A	108.5	C21—C24—H24A	109.5
C13—C12—H12A	108.5	C21—C24—H24B	109.5
C11—C12—H12B	108.5	H24A—C24—H24B	109.5
C13—C12—H12B	108.5	C21—C24—H24C	109.5
H12A—C12—H12B	107.5	H24A—C24—H24C	109.5
C14—C13—C12	106.67 (15)	H24B—C24—H24C	109.5
C14—C13—C17	110.72 (16)	C21—C25—H25A	109.5
C12—C13—C17	109.32 (18)	C21—C25—H25B	109.5
C14—C13—C16	110.46 (19)	H25A—C25—H25B	109.5
C12—C13—C16	111.08 (16)	C21—C25—H25C	109.5
C17—C13—C16	108.59 (17)	H25A—C25—H25C	109.5
C15—C14—C13	113.79 (15)	H25B—C25—H25C	109.5
C15—C14—H14A	108.8	C3—O1—C2	118.21 (15)
C13—C14—H14A	108.8	C15—O3—H3A	109.5
C15—C14—H14B	108.8	C19—O4—H4A	109.5
C13—C14—H14B	108.8		
			
O1—C3—C4—C5	−179.1 (2)	C9—C10—C15—O3	8.8 (3)
C8—C3—C4—C5	1.7 (3)	C11—C10—C15—C14	9.3 (3)
C3—C4—C5—C6	−0.3 (3)	C9—C10—C15—C14	−172.57 (17)
C4—C5—C6—C7	−1.3 (3)	C13—C14—C15—O3	−157.30 (18)
C4—C5—C6—C9	−175.17 (19)	C13—C14—C15—C10	24.0 (3)
C5—C6—C7—C8	1.5 (3)	C10—C9—C18—C19	−79.6 (2)
C9—C6—C7—C8	175.01 (18)	C6—C9—C18—C19	54.6 (2)
C6—C7—C8—C3	−0.1 (3)	C10—C9—C18—C23	95.19 (18)
C4—C3—C8—C7	−1.6 (3)	C6—C9—C18—C23	−130.60 (16)
O1—C3—C8—C7	179.2 (2)	C23—C18—C19—O4	−172.22 (15)
C5—C6—C9—C18	−174.17 (16)	C9—C18—C19—O4	2.6 (3)
C7—C6—C9—C18	12.5 (2)	C23—C18—C19—C20	6.6 (2)
C5—C6—C9—C10	−39.5 (2)	C9—C18—C19—C20	−178.58 (15)
C7—C6—C9—C10	147.15 (18)	O4—C19—C20—C21	−152.94 (16)
C18—C9—C10—C15	−89.34 (19)	C18—C19—C20—C21	28.2 (2)
C6—C9—C10—C15	135.45 (16)	C19—C20—C21—C24	69.2 (2)
C18—C9—C10—C11	88.71 (18)	C19—C20—C21—C22	−52.3 (2)
C6—C9—C10—C11	−46.5 (2)	C19—C20—C21—C25	−171.51 (17)
C15—C10—C11—O2	166.58 (16)	C24—C21—C22—C23	−75.1 (2)
C9—C10—C11—O2	−11.5 (2)	C20—C21—C22—C23	45.6 (2)
C15—C10—C11—C12	−11.6 (2)	C25—C21—C22—C23	165.22 (16)
C9—C10—C11—C12	170.32 (15)	C19—C18—C23—O5	165.19 (16)
O2—C11—C12—C13	162.12 (15)	C9—C18—C23—O5	−10.0 (2)
C10—C11—C12—C13	−19.6 (2)	C19—C18—C23—C22	−13.8 (2)
C11—C12—C13—C14	49.0 (2)	C9—C18—C23—C22	171.03 (15)
C11—C12—C13—C17	168.76 (16)	C21—C22—C23—O5	166.69 (16)
C11—C12—C13—C16	−71.4 (2)	C21—C22—C23—C18	−14.3 (2)
C12—C13—C14—C15	−50.8 (2)	C4—C3—O1—C2	8.4 (3)
C17—C13—C14—C15	−169.63 (19)	C8—C3—O1—C2	−172.5 (2)
C16—C13—C14—C15	70.0 (2)	C1—C2—O1—C3	176.6 (2)
C11—C10—C15—O3	−169.30 (16)		

### Hydrogen-bond geometry (Å, º)

**Table:**

*D*—H···*A*	*D*—H	H···*A*	*D*···*A*	*D*—H···*A*
O3—H3*A*···O5	0.82	1.83	2.631 (2)	164
O4—H4*A*···O2	0.82	1.78	2.5864 (19)	167
